# Supplementary material for: The causal relationship between the human gut microbiota and pyogenic arthritis: a Mendelian randomization study
Source: Front Cell Infect Microbiol. 2024 Nov 26;14:1452480. doi: 10.3389/fcimb.2024.1452480 (PMC11629706; doi:10.3389/fcimb.2024.1452480)
Supplement: Supplementary file 1 [file DataSheet1.zip › mendelian test/ebi-a-GCST90027536.csv_leave_one_out.pdf]

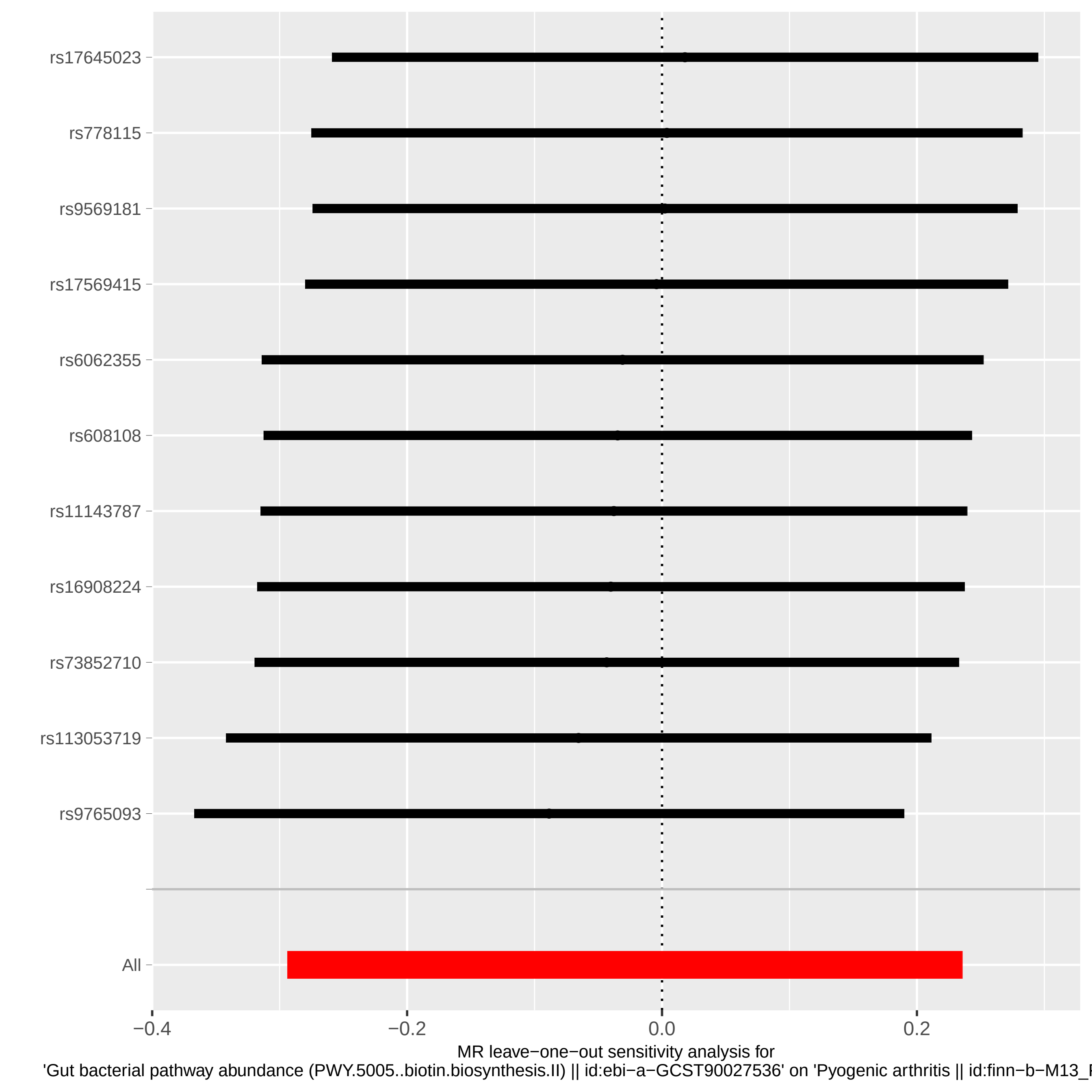
rs17645023
rs778115
rs9569181
rs17569415
rs6062355
rs608108
rs11143787
rs16908224
rs73852710
rs113053719
rs9765093
All
−0.4
−0.2
0.0
0.2
MR leave−one−out sensitivity analysis for
'Gut bacterial pathway abundance (PWY.5005..biotin.biosynthesis.II) || id:ebi−a−GCST90027536' on 'Pyogenic arthritis || id:finn−b−M13_
